# Treatment of Cerebral Ischemia Through NMDA Receptors: Metabotropic Signaling and Future Directions

**DOI:** 10.3389/fphar.2022.831181

**Published:** 2022-02-21

**Authors:** Yuanyuan Li, Xiaokun Cheng, Xinying Liu, Le Wang, Jing Ha, Zibin Gao, Xiaoliang He, Zhuo Wu, Aibing Chen, Linda L. Jewell, Yongjun Sun

**Affiliations:** ^1^ Department of Pharmacy, Hebei University of Science and Technology, Shijiazhuang, China; ^2^ Institute for the Development of Energy for African Sustainability, University of South Africa, Pretoria, South Africa; ^3^ Department of Chemical Engineering, University of South Africa, Florida, South Africa; ^4^ Department of Pharmaceutical Engineering, Hebei Chemical & Pharmaceutical College, Shijiazhuang, China; ^5^ New Drug Research & Development Co., Ltd., North China Pharmaceutical Group Corporation, Shijiazhuang, China; ^6^ Hebei Technological Innovation Center of Chiral Medicine, Shijiazhuang, China; ^7^ Hebei Research Center of Pharmaceutical and Chemical Engineering, Hebei University of Science and Technology, Shijiazhuang, China; ^8^ State Key Laboratory Breeding Base—Hebei Province Key Laboratory of Molecular Chemistry for Drug, Shijiazhuang, China; ^9^ College of Food Science and Biology, Hebei University of Science and Technology, Shijiazhuang, China; ^10^ Department of Pharmacy, Huashan Hospital, Fudan University, Shanghai, China; ^11^ College of Chemical and Pharmaceutical Engineering, Hebei University of Science and Technology, Shanghai, China; ^12^ Department of Chemical Engineering, University of South Africa, Pretoria, South Africa

**Keywords:** NMDA receptor, ion-flow independent, metabotropic signaling, cerebral ischemia, NMDA receptor antagonists

## Abstract

Excessive activation of N-methyl-d-aspartic acid (NMDA) receptors after cerebral ischemia is a key cause of ischemic injury. For a long time, it was generally accepted that calcium influx is a necessary condition for ischemic injury mediated by NMDA receptors. However, recent studies have shown that NMDA receptor signaling, independent of ion flow, plays an important role in the regulation of ischemic brain injury. The purpose of this review is to better understand the roles of metabotropic NMDA receptor signaling in cerebral ischemia and to discuss the research and development directions of NMDA receptor antagonists against cerebral ischemia. This mini review provides a discussion on how metabotropic transduction is mediated by the NMDA receptor, related signaling molecules, and roles of metabotropic NMDA receptor signaling in cerebral ischemia. In view of the important roles of metabotropic signaling in cerebral ischemia, NMDA receptor antagonists, such as GluN2B-selective antagonists, which can effectively block both pro-death metabotropic and pro-death ionotropic signaling, may have better application prospects.

## Introduction

Glutamate receptors mediate glutamate’s excitatory role in physiological processes such as memory, learning, and synaptic plasticity (Hansen et al., 2021); thus, they also play a part in several common neurological diseases, such as depression (Xia et al., 2021), Alzheimer’s disease (Srivastava et al., 2020) and epilepsy (Alcoreza et al., 2021). Glutamate receptors are both ionotropic and metabotropic. The ionotropic N-methyl-d-aspartate (NMDA) glutamate receptor is a tetrameric complex containing two obligatory GluN1 subunits and two additional subunits, either GluN2 (GluN2A-D) or GluN3 (GluN3A-B) (Sun et al., 2019). The diversity of NMDA receptor subtypes endows the receptor family with a variety of physiological and pathological functions (Paoletti et al., 2013; Perez-Otano et al., 2016).

The traditional view on signal transduction through ionotropic glutamate receptors (NMDA receptors, α-amino-3-hydroxy-5-methyl-4-isoxazolepropionic acid (AMPA) receptors, and kainate (KA) receptors) is that glutamate binding opens ion channels, which allow Na^+^, K^+^, or Ca^2+^ to enter or exit the cell and subsequently transmit ion-dependent excitatory signaling (Rajani et al., 2020). However, the discovery of the metabotropic action of KA receptors in 1998 revealed another mode of signal transduction (Rodriguez-Moreno and Lerma, 1998). The metabotropic activities of both KA receptors and AMPA receptors have been found to modulate neurotransmitter release (Falcon-Moya and Rodriguez-Moreno, 2021). With the deepening of research into this subject, there is increasing evidence that NMDA receptors can also mediate both ionotropic and metabotropic signaling (Dore et al., 2016; Dore et al., 2017; Montes De Oca Balderas, 2018). Metabotropic NMDA receptor signaling, which is independent of ion flow, is involved in long-term depression (LTD) (Nabavi et al., 2013), synaptic depression induced by β-amyloid (Aβ) (Kessels et al., 2013; Tamburri et al., 2013; Birnbaum et al., 2015), dendritic spine shrinkage (Stein et al., 2015; Stein et al., 2020) and long-term potentiation (LTP)-induced spine growth (Stein et al., 2021). Recent studies have found that ion-independent metabotropic NMDA receptor signaling plays an important role in the regulation of cerebral ischemic injury (Weilinger et al., 2016; Chen et al., 2017). Metabotropic NMDA receptor signaling has not been found in some other important processes, such as spike timing-dependent plasticity (Rodriguez-Moreno and Paulsen, 2008; Banerjee et al., 2014; Andrade-Talavera et al., 2016) and presynaptic glutamate release modulation (Abrahamsson et al., 2017; Prius-Mengual et al., 2019). This mini review provides a discussion on how metabotropic transduction is mediated by the NMDA receptor, known related signaling molecules, and their interplay in cerebral ischemia.

## NMDA Receptor Metabotropic Operation

The prevailing view on NMDA receptors states that agonist glutamate and co-agonist glycine (or d-serine) jointly activate the receptor, initiating excitatory signaling. Unlike this classical mode, transduction of metabotropic NMDA receptor signaling only requires ligand binding to either one of the two agonist-binding sites, the one for glutamate, GluN2, or the one for glycine, GluN1 (Rajani et al., 2020). By measuring Förster resonance energy transfer (FRET) between fluorescently tagged GluN1 subunits of NMDA receptors, Malinow et al. demonstrated that NMDA exposure induced conformational changes in the cytoplasmic domain of NMDA receptors, provoking synaptic inhibition (Aow et al., 2015; Dore et al., 2015). This phenomenon can be blocked by the glutamate-binding site antagonist amino-phosphonovalerate (APV), but not by the glycine-binding site antagonist 7-chlorokynurenate (7CK) (Aow et al., 2015; Dore et al., 2015). Low-frequency stimulation (LFS) in acute hippocampal slices was shown to induce ion-independent and NMDA receptor-dependent LTD, which could be blocked by the glutamate-binding site antagonist d-amino-phosphonovalerate (D-APV), but not 7CK (Nabavi et al., 2013). In calcium-free extracellular solutions with calcium chelator EGTA or BAPTA, glycine exposure increased the level of Akt phosphorylation in cultured mouse cortical neurons, which was inhibited by the glycine-binding site antagonist, L-689560, and the addition of NMDA receptor ion-channel blocker, MK-801 or GluN2B-selective antagonist, Ro 25-6981 could not prevent this effect (Hu et al., 2016).

Similar to non-channel transmembrane receptors, agonist-induced conformational change in the cytoplasmic domain of NMDA receptors is a key requirement for metabotropic signaling transduction. Using the FRET technique, Dore et al. showed that in the presence of 7CK or MK-801, FRET between different GluN1 subunits on individual NMDA receptors could be reduced after NMDA was administered, which indicated that the binding of NMDA to NMDA receptors causes conformational changes in the cytoplasmic domain in the absence of ion flow (Dore et al., 2015). Intracellular infusion of a GluN1 C-terminus antibody that can bind and immobilize two nearby cytoplasmic domains of the GluN1 subunit prevented FRET changes induced by NMDA exposure (Dore et al., 2015).

The relative position change and resulting interaction between different molecules coupled to the C-terminus of NMDA receptors induced by conformational changes are the underlying molecular mechanisms of metabotropic NMDA signaling transduction. Studies have shown that both protein phosphatase 1 (PP1) and calcium/calmodulin-dependent protein kinase II (CaMKII) bind to the intracellular C-terminus of NMDA receptors (Aow et al., 2015; Sun et al., 2018). Without ligands binding to NMDA receptors, the distance between PP1 and CaMKII is too large for any interaction to occur. However, when NMDA binds to NMDA receptors, the relative positions of PP1 and CaMKII change, and the distance between them is reduced. In this situation, the catalytic site of PP1 can contact CaMKII, and dephosphorylate it at Thr286 (Aow et al., 2015). Thereafter, CaMKII is repositioned on the NMDA receptor and subsequently activates downstream signaling molecules, thereby inducing synaptic inhibition in an ion-independent manner (Aow et al., 2015).

Although it is independent of ion transmembrane flow, metabotropic NMDA receptor signaling may require the involvement of intracellular calcium and its effectors. Studies have indicated that the metabotropic actions of KA receptors are involved in modulating glutamate release in a biphasic manner (Falcon-Moya and Rodriguez-Moreno, 2021). KA receptor-mediated facilitation of glutamate release is dependent on Ca^2+^, calmodulin, and protein kinase A (PKA) (Andrade-Talavera et al., 2012; Andrade-Talavera et al., 2013; Falcon-Moya et al., 2018; Falcon-Moya and Rodriguez-Moreno, 2021). KA receptor-mediated depression of glutamate release is dependent on Ca^2+^, calmodulin, protein kinase A (PKA), and G-protein (Falcon-Moya et al., 2018; Falcon-Moya and Rodriguez-Moreno, 2021). Whether these signaling molecules are involved in metabotropic NMDA receptor-mediated actions should be studied in the future.

## Signaling Molecules Mediating Metabotropic NMDA Receptor Signaling

Metabotropic NMDA receptor actions involve signaling molecules, such as kinases, second messengers, and other molecules that have been found to be related to synaptic plasticity and cerebral ischemia (Table 1).

**TABLE 1:** Downstream signaling molecules of metabotropic NMDA receptor signaling.

Pathophysiological processes	Related subunits	Downstream signaling molecule	References
Spine shrinkage	Not reported	nNOS, NOSIAP, p38, MK2, cofilin	Nabavi et al. (2013); Stein et al. (2020)
		CaMKII	
Synaptic depression	GluN2	p38	Stein et al. (2020)
LTD	GluN2	p38	Nabavi et al. (2013); Birnbaum et al. (2015)
	Not reported	PP1, CaMKII	Coultrap et al. (2014); Aow et al. (2015)
LTP	Not reported	CaMKII	Coultrap et al. (2014)
Enhance the function of the AMPA receptor	GluN2A	ERK1/2	Li et al. (2016)
Excitotoxic injury	GluN1, GluN2A	Akt	Hu et al. (2016)
	GluN1	Src, Panx1	Weilinger et al. (2012); Weilinger et al. (2016)
	GluN2B	PI3K, NOX2	Minnella et al. (2018)

### Signaling Molecules Related to Synaptic Plasticity

Neuronal nitric oxide synthase (nNOS)/nitric oxide synthase one adaptor protein (NOS1AP)/p38/MAPK-activated protein kinase 2 (MK2)/cofilin is a key metabotropic NMDA receptor signaling pathway for gating the structural plasticity of dendritic spines. nNOS is a member of the NMDA receptor complex that anchors to the scaffold protein postsynaptic density-95 (PSD-95) (Sun et al., 2015). NOS1AP is a carboxy-terminal ligand of nNOS (Zhu et al., 2020). L-TAT-GESV, an uncoupling agent of the nNOS/NOS1AP complex (Li et al., 2013), interferes with dendritic spine shrinkage driven by metabotropic NMDA receptor signaling (Stein et al., 2020). The NOS inhibitor l-NNA was shown to abolish high-frequency uncaging (HFU)-induced NMDA receptor-dependent spine shrinkage mediated by non-ionotropic signaling (Stein et al., 2020). p38, MK2, and cofilin are specific downstream signaling molecules of NOS1AP (Stein et al., 2020). Interestingly, during strong Ca^2+^ influx following LTP induction, this signaling pathway promotes spine growth (Stein et al., 2021). It is still unclear how metabotropic NMDA receptor signaling affects nNOS. Although nNOS is a member of the NMDA receptor complex, it may play a physiological role in an NMDA receptor-independent manner. For example, nNOS-derived NO is involved in the recently discovered developmental switch from an NMDA receptor-dependent form of spike timing-dependent LTD to NMDA receptor-independent LTP (Falcon-Moya et al., 2020).

PP1 and CaMKII are two important downstream signaling molecules of metabotropic NMDA receptor signaling involved in the process of synaptic depression. PP1 becomes an indirect coupling molecule of the GluN1 subunit by binding to yotiao (Westphal et al., 1999). CaMKII is a direct binding partner of GluN2 subunits. Both residues 1120–1482 or residues 839–1120 in GluN2B and the 1389–1464 sequence in the C-terminus of GluN2A are sufficient for the binding of CaMKII (Sun et al., 2018). NMDA binding was shown to produce a transient change in the relative position between PP1 and CaMKII, allow PP1 to act on CaMKII and dephosphorylate CaMKII at Thr286 (Aow et al., 2015). This change induced a reorientation of CaMKII within the C-terminus of NMDA receptors and caused CaMKII to potentially catalyze substrates necessary for LTD (Aow et al., 2015).

p38 is also involved in synaptic depression mediated by metabotropic NMDA receptor signaling. NMDA exposure increased p38 phosphorylation in cultured neurons, which could be blocked by D-APV but not by MK-801 (Nabavi et al., 2013). Synaptic depression can be induced by Aβ exposure, and the p38 inhibitor SB239063 abolishes this phenomenon (Birnbaum et al., 2015). Because p38 is not a member of the NMDA receptor complex, further studies are needed to identify the related upstream signaling molecules.

Extracellular signal-regulated kinase 1/2 (ERK1/2) participates in the transduction of metabotropic NMDA receptor signaling. Co-incubation of hippocampal slices with metabotropic glutamate receptor type 5 (mGluR5) agonist CHPG (15 μΜ) and NMDA (5 μΜ) induced a robust increase in the phosphorylation level of ERK1/2, which could be inhibited by AP5, but not by MK-801 (Krania et al., 2018). This phenomenon could also be prevented by the Src inhibitor PP1, which indicated the involvement of Src in this process (Krania et al., 2018). Glycine increased ERK1/2 phosphorylation in a dose-dependent manner, in hippocampal neurons exposed to a Ca^2+^-free extracellular solution with EGTA, MK-801, and strychnine (Li et al., 2016). This effect of glycine appeared in HEK293 cells transfected with cDNAs of GluN1 and GluN2A, but not in cells transfected with cDNAs of GluN1 and GluN2B (Li et al., 2016).

### Signaling Molecules Related to Cerebral Ischemia

The NMDA receptor, Src, and pannexin 1 (Panx1) comprise a metabotropic signaling complex that is involved in the process of cerebral ischemia (Li et al., 2021). Src indirectly associates with NMDA receptors by interacting with NADH dehydrogenase subunit 2 (ND2) via amino acids 40–80 (Gingrich et al., 2004; Liu et al., 2008; Sun et al., 2016). Src is anchored to NMDA receptors through the interaction between the PDZ3 domain of PSD-95 and the SH2 domain of Src (Kalia and Salter, 2003; Sun et al., 2016). Panx1 interacts with Src via the amino acid sequence 305–318 at the C terminus (Weilinger et al., 2012). The relative amount of Src associated with the NMDA receptor complex increased following NMDA and glycine exposure, and the phosphorylation level at Tyr416 also increased (Weilinger et al., 2016). Src can open Panx1 channels by phosphorylating Panx1 at Tyr308, which can be prevented by the SFK inhibitor PP2 (Weilinger et al., 2012; Weilinger et al., 2016). NMDA receptor competitive antagonists APV plus CGP-78608, but not MK-801, can prevent NMDA-induced Panx1 currents (Weilinger et al., 2016).

Akt is another downstream metabotropic signaling molecule involved in cerebral ischemia. In a modified calcium-free extracellular solution with EGTA or BAPTA, treating mouse cortical neurons with glycine significantly enhanced the activity of Akt, which could be blocked by L-689560, but not by MK-801 or the glycine receptor antagonist, strychnine (Hu et al., 2016). After inhibiting ion flow by NMDA receptors, glycine exposure increased Akt phosphorylation level in GluN1/GluN2A transfected HEK293 cells, but not in GluN1/GluN2B-transfected cells (Hu et al., 2016). This indicates that glycine can enhance Akt phosphorylation through the metabotropic signaling of NMDA receptors containing GluN2A. Similarly, glycine could also reduce the infarct volume in the brain of ischemic stroke rats pre-injected with MK-801 and strychnine; this effect was sensitive to L-689560 and Akt inhibitor IV (Chen et al., 2017).

In addition to participating in the regulation of synaptic plasticity, p38 is involved in neuronal damage induced by cerebral ischemia. p38 activation induced by glutamate exposure or NO donors contributes to excitotoxic neuronal cell death (Cao et al., 2005). The nNOS-PBD (PSD95-binding domain) construct containing the nNOS PDZ domain and the adjacent β finger, which binds PSD95 in a manner similar to nNOS, reduced p38 activation and decreased glutamate-induced pyknosis in neurons (Cao et al., 2005). The NMDA receptor-PSD-95-nNOS-NOS1AP-MAP kinase 3 (MKK3) is the upstream signaling pathway of p38 (Cao et al., 2005; Li et al., 2013; Sun et al., 2015).

In contrast to previous signaling pathways, NADPH oxidase-2 (NOX2) activation requires both ionotropic and metabotropic NMDA receptor signaling. In mouse cortical neuron cultures, NMDA-induced superoxide production was blocked by the application of 7CK, L-689560, or MK-801, and after additional addition of ionomycin to provide a Ca^2+^ influx, superoxide production was restored (Minnella et al., 2018). However, AP5 prevented NMDA-induced NOX2 activation, and this effect could not be reversed by co-incubation with ionomycin (Minnella et al., 2018). NOX2 does not form a complex with the NMDA receptor. The upstream signaling molecule phosphatidyl-inositol 3-kinase (PI3K) binds to GluN2B via its p85 regulatory subunit (Wang and Swanson, 2020). After NMDA stimulation, the activation of PI3K induces the formation of phosphatidylinositol (3,4,5) trisphosphate (PIP3) and PIP3 activates protein kinase C (PKC) and phosphorylates the p47^phox^ organizing subunit of NOX2 (Brennan-Minnella et al., 2015; Wang and Swanson, 2020).

## Roles of Metabotropic NMDA Receptor Signaling in Cerebral Ischemia

**FIGURE 1:**
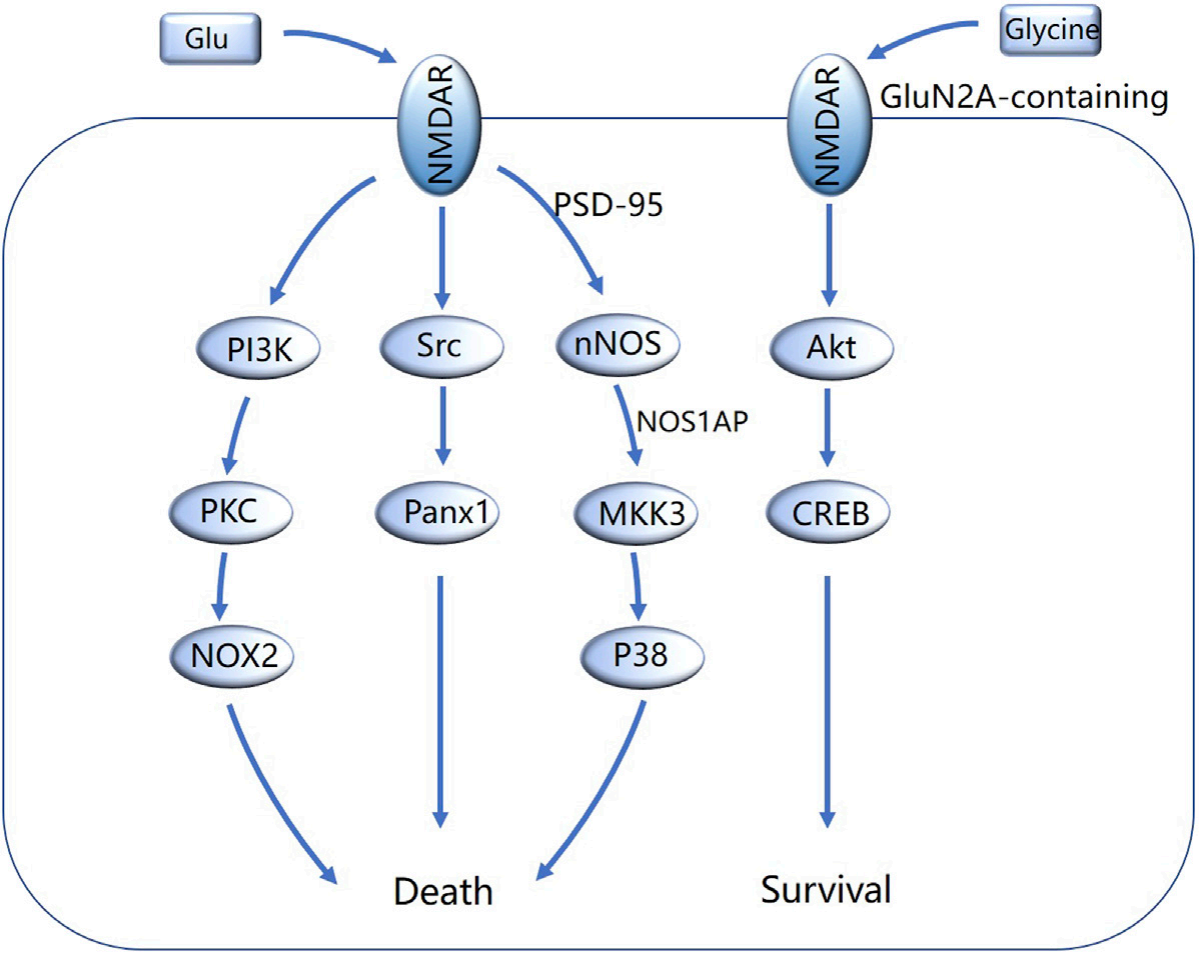
Overview of metabotropic NMDA receptor (NMDAR) signaling pathways involved in cerebral ischemia. Excessive glutamate binds to the GluN2 subunit of NMDA receptors and initiates several pro-death signaling pathways, such as PI3K-PKC-NOX2, Src-PanX1 and nNOS-MKK3-p38. Glycine binds to the GluN1 subunit of GluN2A-containing NMDA receptors, activates Akt-CREB signaling pathway and promotes the survival of neurons.

Metabotropic NMDA receptor signaling regulates the damage induced by cerebral ischemia in a bidirectional manner (Figure 1). In general, metabotropic signaling mediated by GluN2B-containing NMDA receptors plays an important role in promoting neuronal death, whereas GluN2A-containing NMDA receptors play a neuroprotective role.

### Pro-Death Effect

The metabotropic NMDA receptor-Src-Panx1 signaling pathway exerts a pro-death effect in cerebral ischemia. Over-activation of NMDA receptors activates Src, induces phosphorylation of Panx1 at the Tyr308 site, opens the Panx1 half-channel, and ion-independently causes neuronal death (Weilinger et al., 2012; Weilinger et al., 2016). A combination of the competitive glutamate site antagonist APV and glycine site antagonist CGP-78608 blocked the opening of the Panx1 half channel and prevented excitotoxic damage in hippocampal CA1 pyramidal neurons (Weilinger et al., 2016). Polypeptide Src48, which interferes with GluN1-Src interaction, or Tat-Panx308, which interferes with Panx1 phosphorylation, showed a neuroprotective effect *in vitro* (Weilinger et al., 2016). In an *in vivo* model of stroke, Tat-Panx308 reduced infarction volume by approximately 9.7% (Weilinger et al., 2016).

The NMDA receptor-PI3K-PKC-NOX2 is a pro-death metabotropic NMDA receptor signaling pathway. NOX2 is the primary source of neuronal superoxide production in response to NMDA receptor activation (Brennan-Minnella et al., 2015; Minnella et al., 2018). Superoxide production largely contributes to neuronal death during excitotoxicity following cerebral ischemia (Brennan-Minnella et al., 2015). The signaling pathway that links NMDA receptors to NOX2 activation as well as superoxide production is triggered by NMDA binding, but not glycine binding, which can be blocked by the glutamate-binding site antagonist AP5 (Minnella et al., 2018; Wang and Swanson, 2020). Neurons deficient in GluN2B or expressing chimeric GluN2B/GluN2A C-terminus subunits did not exhibit NMDA-induced superoxide production, indicating that GluN2B-containing NMDA receptors are preferentially involved in NMDA-induced superoxide production (Minnella et al., 2018).

p38 may also be a downstream pro-death metabotropic signaling molecule of NMDA receptors during cerebral ischemia. p38 is strongly involved in excitotoxicity, and the cell-permeable peptide, TAT-GESV effectively inhibits excitotoxic p38 activation, which protects against excitotoxic neuronal damage and reduces ischemic injury in neonatal hypoxia-ischemia rats (Li et al., 2013). NMDA exposure in cultured neurons activates p38 in an ion-independent manner (Nabavi et al., 2013).

### Pro-Survival Effect

The metabotropic NMDA receptor signaling mediated by GluN2A may play a neuroprotective role in cerebral ischemia. Glycine administration reduced infarct volume in middle cerebral artery occlusion (MCAO) animals pretreated with MK-801 and strychnine; this effect was sensitive to glycine site antagonists and can also be blocked by Akt inhibitors (Chen et al., 2017). After inhibiting ion flow by NMDA receptors, glycine exposure increased Akt phosphorylation level in GluN1/GluN2A transfected HEK293 cells, but not in GluN1/GluN2B-transfected cells (Hu et al., 2016). This indicates that glycine can enhance Akt phosphorylation through the metabotropic signaling mediated by NMDA receptors containing GluN2A.

## Future Directions of NMDA Receptor Antagonists

The roles of NMDA receptors in cerebral ischemia are complex. NMDA receptors mediate both pro-death and pro-survival ionotropic signaling. Similarly, the metabotropic signaling of NMDA receptors can either be beneficial or harmful to neuronal survival. This makes the design of effective treatment strategies based on NMDARs difficult. The complexity of NMDA receptor signaling may be one of the important underlying reasons for the failure of NMDA receptor antagonists in the treatment of cerebral ischemia. Researchers should study how to effectively block all pro-death ionotropic and pro-death metabotropic signaling. Among all NMDA receptor antagonists, ion-channel blockers and glycine-binding site antagonists cannot block pro-death metabotropic signaling. Although glutamate-binding site antagonists can inhibit both ionotropic and metabotropic signaling, they have no selectivity for GluN2A and GluN2B. In theory, GluN2B-selective antagonists may have unique advantages for blocking the pro-death effect of both ionotropic and metabotropic signaling without influencing the pro-survival effect of GluN2A. However, existing GluN2B-selective antagonists are negative allosteric regulators and have the disadvantages of off-target effects and activity dependence (Kew et al., 1996; Fischer et al., 1997; Dey et al., 2016). GluN2B-selective glutamate-binding site antagonists may be a promising research and development direction for NMDAR antagonists.
